# Shared biological mechanisms of depression and obesity: focus on adipokines and lipokines

**DOI:** 10.18632/aging.204847

**Published:** 2023-06-29

**Authors:** Xiying Fu, Yicun Wang, Fangyi Zhao, Ranji Cui, Wei Xie, Qianqian Liu, Wei Yang

**Affiliations:** 1 Department of Endocrinology, The Second Hospital of Jilin University, Changchun 130041, P.R. China; 2 Jilin Provincial Key Laboratory for Molecular and Chemical Genetics, The Second Hospital of Jilin University, Changchun 130041, P.R. China; 3 Department of Neurology, The Second Hospital of Jilin University, Changchun 130041, P.R. China

**Keywords:** depression, obesity, neuroendocrine mechanism, inflammation, neuroplasticity

## Abstract

Depression and obesity are both common disorders currently affecting public health, frequently occurring simultaneously within individuals, and the relationship between these disorders is bidirectional. The association between obesity and depression is highly co-morbid and tends to significantly exacerbate metabolic and related depressive symptoms. However, the neural mechanism under the mutual control of obesity and depression is largely inscrutable. This review focuses particularly on alterations in systems that may mechanistically explain the *in vivo* homeostatic regulation of the obesity and depression link, such as immune-inflammatory activation, gut microbiota, neuroplasticity, HPA axis dysregulation as well as neuroendocrine regulators of energy metabolism including adipocytokines and lipokines. In addition, the review summarizes potential and future treatments for obesity and depression and raises several questions that need to be answered in future research. This review will provide a comprehensive description and localization of the biological connection between obesity and depression to better understand the co-morbidity of obesity and depression.

## INTRODUCTION

Obesity is a long-term metabolic disease triggered in part by the mutual interaction of genetic, environmental as well as other factors. According to WHO criteria, obesity is commonly described in terms of body mass index (BMI) ≥ 30 kg/m^2^ [1, 2]. Obesity is associated with an elevated risk of depression, metabolic, anxiety, cardiovascular, and chronic inflammation, as well as some malignant diseases [3]. Studies have shown that obesity is most likely to amplify the prevalence of depression and anxiety disorders [4, 5]. Obesity stems from an expansion of adipose tissue and an imbalance between caloric intake and energy expenditure [6]. Obese patients with higher amounts of visceral fat are at higher risk for serious complications [7]. White adipocytes, which store energy, and thermogenic brown and beige adipocytes, which produce energy, secrete hormones, such as adipokines, lipokines and exosomal microRNAs [8]. The secretion of adipokines and lipokines, such as leptin, adiponectin, monocyte chemoattractant protein-1 (MCP-1), plasminogen activator inhibitor type 1 (PAI-1), retinol Binding Protein 4 (RBP4), visfatin, resistin, apelin, chemerin, palmitoleic acid and lysophosphatidic acid is modified in the presence of adipose tissue dysfunction and may cause a range of obesity-related disorders [9–11]. With a greater comprehension of the functional and molecular targets of adipokines and lipokines, it will hold enormous promise for both new drug treatment strategies and diagnostics.

Depression is a severe psychiatric disorder characterized by persistent low mood, diminished interest, slowed thinking, reduced volitional activity, sleep difficulties or disturbances in appetite, affecting more than 300 million people worldwide and the number is growing [12, 13]. There is a growing body of research recognizing that negative emotions such as anxiety, stress and depression exert a significant negative influence on health and illness. There are also relevant clinical studies that show that obesity suffers from varying degrees of depressive symptoms [14, 15]. Recent scientific evidence suggests that depression and obesity are not independently linked. The two disorders can be interrelated through a vicious, mutually reinforcing cycle of maladaptive physiological adaptations [5]. Lassale et al. demonstrated a bidirectional and complex multifactorial relation between mood disorders and obesity [16]. Data from their meta-analysis revealed that men and women suffering from obesity had a 55% elevated risk of developing depression, while those suffering from depression had a 58% higher risk of developing obesity [16]. There is growing evidence that adipocytokine and lipokine levels are up- or down-regulated in the progression of depression [10, 11, 17]. Furthermore, a large body of evidence suggests that modulating adipocytokine and lipokine levels in models of depression attenuates or promotes depressive-like behaviors [18–20]. Currently, the pathogenesis of depression is not completely clear. There are many hypotheses for depression, such as the monoamine neurotransmitter hypotheses, neurotrophic factor hypotheses (mainly brain-derived neurotrophic factor (BDNF) expression and functional downregulation), neurocircuitry hypotheses, neuroendocrine hypotheses, neuroinflammation hypotheses, gut microbiota hypotheses, neuroplasticity hypotheses, hypothalamic-pituitary-adrenal (HPA) axis hypothesis, etc. [21–26].

Among them, neuroendocrine hypotheses, inflammation, gut microbiota, neuroplasticity and abnormal HPA axis function are currently the most popular directions for investigating the pathogenesis of depression. It has been established nowadays that dysregulation of the innate and adaptive immune system happens in patients with depression and that inflammatory processes are highly correlated with the pathophysiology of depression [27]. Studies have suggested that a sustained, low-grade inflammatory response is a potentially modifiable risk factor for obesity [28, 29]. In addition, there is increasing evidence that the microbial community of the entire gastrointestinal tract (gut microbiota) is related to depressive disorders [30]. Multiple lines of evidence suggest that the gut microbiota is participating in the progression of obesity and related co-morbidities [30–36]. The neuroplasticity hypothesis, suggesting that the antidepressant effect can be regulated by modulating synaptic plasticity in hippocampal neurons and thus affecting structural plasticity in neural networks, is also the hypothesis applied in the study of the rapid antidepressant effect of ketamine [21, 37]. High-fat diet (HFD) feeding is currently a common method for establishing obese animal models [38, 39]. Studies have suggested that HFD leads to persistent elevations in cytokines and chemokines that can cause region-specific neuroplasticity, thereby promoting mood deficits and increased body weight [4]. Neuroendocrine studies have suggested that the HPA axis is relevant to the pathophysiology of depression, and studies have confirmed the overactivity of the HPA axis in patients with major depressive disorder (MDD) [40]. Research shows that the HPA axis may influence the body weight of stressed individuals by regulating cortisol [41]. Taken together, this review offers a well-rounded description of the mechanisms underlying the development of obesity and depression to better understand the interrelationship between the two conditions and to provide more effective treatment approaches.

## Shared biological mechanisms of depression and obesity

**Figure 1:**
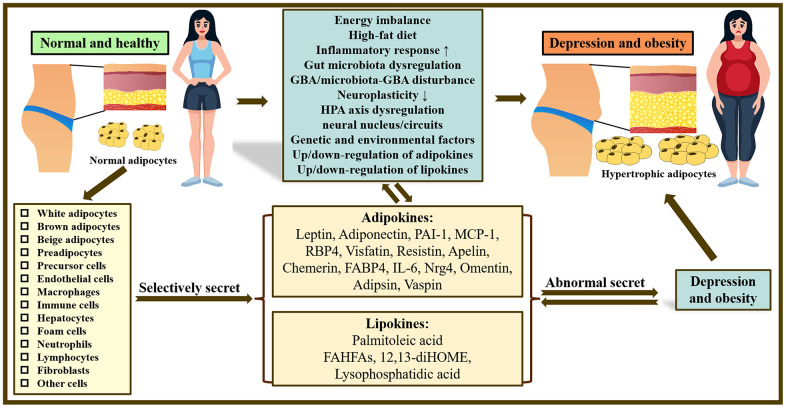
**Overview of the mechanisms underlying the co-pathogenesis of depression and obesity: based on adipokines and lipokines.** In the body, there are many kinds of adipocytes and different adipocytes can selectively release related adipokines and lipokines. Abnormal secretion of adipokines and lipokines plays a crucial role in obesity and depression and may be related to increased inflammatory response, gut microbiota disturbance, neuroplasticity, dysfunction of HPA as well as GBA/microbiota-GBA disturbance. Note: HPA axis, hypothalamic-pituitary-adrenal axis; GBA, gut-brain-axis; PAI-1, Plasminogen activator inhibitor type 1; MCP-1, Monocyte chemoattractant protein-1; RBP4, Retinol Binding Protein 4; FABP4, fatty acid-binding protein 4; IL-6, Interleukin 6; Nrg4, Neuregulin 4; FAHFAs, fatty acid esters of hydroxy fatty acids; 12,13-diHOME, 12,13-dihydroxy-(9Z)-octadecenoic acid.

**Figure 2:**
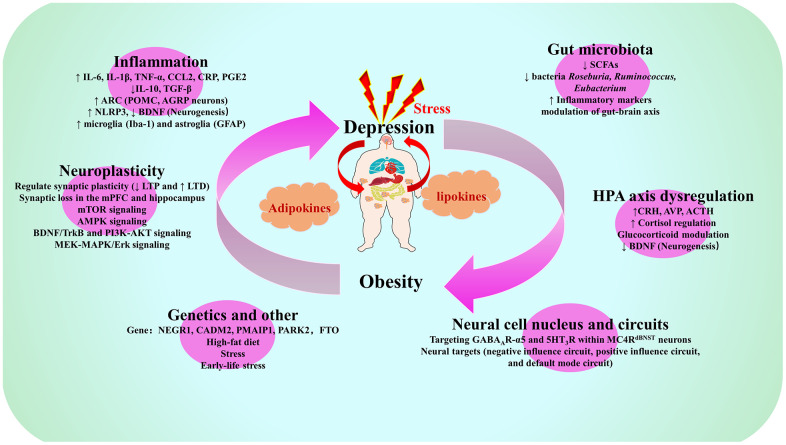
**Summary map of common biological mechanisms of depression and obesity.** Note: IL-6, Interleukin-6; IL-1β, Interleukin-1β; TNF-α, Tumor necrosis factor-α; CCL2, C-C motif chemokine ligand 2; CRP, C-reactive protein; PGE2, prostaglandin E2; IL-10, Interleukin-10; TGF-β, transforming growth factor-beta; ARC, arcuate nucleus of the hypothalamus; POMC, pro-opiomelanocortin; AGRP, agonist peptide-related peptide; NLRP3, NOD-like receptor family pyrin domain containing 3; BDNF. brain derived neurotrophic factor; Iba-1, ionized calcium binding adapter molecule-1; GFAP, glial fibrillary acidic protein; SCFAs, short-chain fatty acids; LTP long-term potentiation, LTD, long-term depression; mPFC, medial prefrontal cortex; mTOR, mechanistic target of rapamycin; AMPK, AMP-activated protein kinase; TrkB, tyrosine kinase receptor B; PI3K, phosphatidylinositol 3-kinase; AKT, protein kinase B; MEK, MAPK/extracellular signal-regulated kinase; MAPK, mitogen-activated protein kinases; ERK, extracellular signal-regulated kinase; CRH, corticotropin-releasing hormone; AVP, arginine vasopressin; ACTH, adrenocorticotropic hormone; NEGR1, neuronal growth regulator 1; CADM2, Cell adhesion molecule 2; PMAIP1, phorbol-12-myristate-13-acetate-induced protein 1; PARK2, E3 ubiquitin-protein ligase parkin; FTO, Fat mass and obesity-associated gene; GABA, gamma-aminobutyric acid; MC4R, melanocortin 4 receptor; dBNST, bed nucleus of the stria terminus.

There is ample reason to assume that depression and obesity are interrelated via a vicious, mutually strengthening cycle of negative physiological adaptations. In this section, we review the mechanisms underlying the co-pathogenesis of obesity and depression, mainly around the inflammation, gut microbiota, gut-brain axis (GBA)/microbiota-gut-brain axis (microbiota-GBA), neuroplasticity and HPA axis abnormalities (Figures 1, 2).

### The potential role of inflammation in depression and obesity

Overactivation of the immune system is caused by several factors, such as difficult life situations, stress from society, and poor lifestyle habits (smoking, lack of exercise, HFD), all of which cause an increased inflammatory response and thus promote depressive symptoms [42]. Elevated levels of inflammatory markers such as IL-1β, IL-6, IL-2, TNF-α, CRP and PGE2 were found in patients with depression [43–47]. The cytokine hypothesis suggests that in depression, the number of pro-inflammatory cytokines such as IL-6, IL-1β and TNF-α is increased, while the number of anti-inflammatory cytokines such as IL-10 and TGF-β is decreased, tilting the overall immune response toward inflammation [48, 49].

The increase in macroglia peripheral nervous system and central nervous system (CNS) cytokines is coupled with the activation of microglia, the brain’s immunocompetent cells. This, in turn, has been linked to changes in synaptic plasticity, neurogenesis and emotional behavior [50]. A variety of key inflammatory markers also have been implicated in the risk of adverse outcomes of obesity and obesity-related diseases [48]. Adipocytes in abdominal, muscle and liver fat produce pro-inflammatory and chemotactic compounds such as IL-6, IL-1β, TNF-α and C-C motif chemokine ligand 2 (CCL2), as well as inflammatory hormones such as adiponectin and leptin [51, 52]. Overweight and obese individuals have adipocytes and macrophages in their adipose tissue that cause the secretion of cytokines and chemokines which can cross the blood-brain barrier (BBB) as well as stimulate neuroinflammation [53, 54].

Neuroinflammation triggered by obesity has recently been demonstrated to influence multiple brain structures like the hippocampus, cortex, brainstem or amygdala, in addition to being linked to an elevated incidence of central disorders including depression as well as impaired cognitive function [55]. Chronic overnutrition and obesity can lead to chronic low-level inflammation across the body, a state of chronic inflammation known as “meta-inflammation” mediated by macrophages located in the colon, liver, muscle as well as adipose tissue [56]. HFD is associated with the production of cytokines by non-neuronal cells in the hypothalamus that activate diverse inflammatory mediators in the arcuate nucleus of the hypothalamus (ARC), a primary region of the hypothalamus that regulates pro-opiomelanocortin (POMC) and agonist peptide-related peptide (AGRP) neurons, the primary neurons that sense and integrate peripheral metabolic signals and respond accordingly [57, 58]. Therefore, it is important to discuss the role of neuronal and non-neuronal cells in HFD-induced hypothalamic inflammation. The vagus nerve is linked to the visceral organs and the CNS, and the vagal afferent nerve conveys hunger signals to the hypothalamus via the gastric-derived orexigenic peptide ghrelin [59]. Studies have shown that intake of HFD triggers inflammatory responses in the gut, nodal ganglia and hypothalamus within a short period vagal afferent nerves can transmit inflammatory signals of gut origin to the hypothalamus through the nodose ganglion, while ghrelin can prevent inflammation induced by HFD [58, 59]. Ingestion of HFD leads to a lack of integrity of the gut barrier, causing the transfer of macromolecules, such as microbial or pathogen-associated molecular patterns (e.g., lipopolysaccharides) entering the systemic circulation [60]. Nucleotide-binding oligomerization domain–like receptor family pyrin domain-containing 3. (NLRP3) inflammatory vesicles are an essential component of the innate immune system, mediating caspase-1 activation and secretion of the pro-inflammatory cytokine IL-1β/IL-18 in response to microbial infection as well as cellular injury [61, 62]. Li et al. showed that microglia NLRP3 inflammatory vesicles induce neurotoxic astrocyte function by activating the neuroinflammatory caspase-1 pathway as a response to chronic stress [63]. Vandanmagsar et al. showed that NLRP3 inflammatory vesicles sense danger signals associated with obesity and lead to obesity-induced inflammation and insulin resistance [51]. The study by Schachter et al. suggests that reducing obesity-related neuroinflammation may have beneficial effects on depression [30]. Li et al. revealed that HFD-induced diabetes and obesity-related neuroinflammation activated the transcription factor CCAAT/enhancer binding protein beta (C/EBPβ) in hippocampal neurons and that this factor suppressed BDNF expression and caused depression-like behavior in male mice [64]. This suggests that inflammation-activated neuronal C/EBPβ can contribute to HFD-induced depression by decreasing BDNF expression [53].

BDNF is mainly produced by microglia and astrocytes and has neuroprotective as well as anti-inflammatory effects [65, 66]. Obesity creates an environment of chronic inflammation that leads to negative physiological and neurological outcomes such as diabetes, cardiovascular disease, and depression. Although the entire body is involved in metabolic homeostasis, the neuroimmune system has recently emerged as a key regulator of metabolism [67]. Lam et al. found that 12 weeks of HFD feeding not only led to obesity in mice but also to depression-like behaviors and that astrocyte activation, which is closely associated with depression, was also evident in the ventral hippocampus [68]. Four weeks of pioglitazone treatment attenuated HFD-induced glucose metabolic dysfunction, upregulated ventral hippocampal GFAP (glial fibrillary acidic protein), reduced the total process length and number of branching sites of hippocampus CA1 GFAP-immunoreactive astrocytes in the ventral hippocampus, and attenuated the depressive phenotype, suggesting that pioglitazone may be a promising therapeutic agent for metabolic disorders and related depression [68]. Microglia are resident immune cells in the brain that respond directly and indirectly to dietary fat, and the environment in which microglia develop helps them to respond later in life [67]. Numerous studies have shown that chronic peripheral inflammatory states reduce adult hippocampal neurogenesis [69–71]. Peripherally released pro-inflammatory cytokines are implicated in communicating between the peripheral immune system and the brain via the activation of microglia in the brain [71]. Activated microglia may reduce neurogenesis by inhibiting the proliferation of neural stem cells, increasing apoptosis of neural progenitors, and decreasing the survival of newly developing neurons as well as their integration into existing neural circuits [69]. Stress-induced microglia activation and neuroinflammation play a vital role in the pathogenesis of depression [72, 73]. However, obese patients with chronic low-grade inflammation are at increased risk of developing depression [74]. Peroxisome proliferator-activated receptor gamma (PPARγ) is a nuclear transcription factor that moderates microglia polarization and neuroinflammation [72]. Qin et al. showed that chronic unpredictable mild stress (CUMS) was able to induce severe depression-like behavior, neuroinflammation and reduced PPARγ expression in leptin-deficient (ob/ob) mice compared to wild-type/C57BL/6J mice, suggesting that PPARγ mediates an activated phenotype of microglia, which may be related to the susceptibility of stressed ob/ob mice to develop depression [72].

### The potential role of gut microbiota in depression and obesity

The degree of diversity and balance of gut microbiota strains is an essential indicator of the overall health status of the organism [75]. Depression and obesity have a high comorbidity in individuals, which may be due to sharing similar risk factors, including disruption of the gut microbiota. The gut microbiota is composed of over 100 trillion microorganisms, including a minimum of 1000 various species of bacteria, and plays a crucial role in the physiological and pathophysiological processes that occur in the body [76, 77]. The gut microbiota evolved together with the host and is an indivisible part of the body. Gut microbes are diverse and affect many processes, including immunity, metabolism as well as the CNS. Microbiota-GBA has emerged as a new candidate for depression [78, 79]. It has been shown that patients with depression, which includes those in remission, have a various gut microbiota composition than healthy controls [80]. Animal models of depression have shown changed gut microbiota compared to controls [81]. In a further meta-analysis, disturbances in the gut were found to be correlated with a decrease in certain anti-inflammatory butyrate-producing bacteria as well as an increased number of pro-inflammatory bacteria in patients with depression and other psychiatric disorders [82]. The fat mass and obesity associated gene (FTO) [83], an RNA demethylase [84, 85], is downregulated in the hippocampus of patients with MDD and in mouse models of depression [86]. Suppression of Fto expression in the mouse hippocampus results in depression-like behaviour in adult mice, whereas overexpression of FTO expression rescues the depression-like phenotype, suggesting that FTO is a modulator of depression-like behavioural mechanisms in mice [86]. Sun et al. showed that Fto deletion resulted in decreased body weight, reduced anxiety and depression-like behavior, and reduced sensitivity to stressful stimuli in Fto+/- mice [36]. In terms of intestinal flora, Fto-deficient mice were characterized by specific anti-inflammatory bacteria, importantly, the behavioral changes in Fto+/- mice were mediated by changes in the gut microbiota [36]. The results on the role of FTO in depression-obesity comorbidity are inconclusive, and further research is needed to deepen knowledge of the genetic basis of this comorbidity [87].

It has been shown that dysregulation of the gut microbiota in mice is associated with several neurobiological traits of depression, such as mild chronic inflammation, abnormal activity of the HPA axis, and reduced adult neurogenesis [88, 89] Recent studies suggest that chronic inflammation resulting from a HFD may exert a central role in the induction of neuroinflammation and depression [30]. Remarkably, the gut microbiota mediates some of the effects of HFD on human physiology and influences host mood and behavior. Pathogens, stressors, and predisposing factors can all contribute to excessive or protracted inflammatory responses (moderators include childhood trauma and obesity). The ensuing illness behaviors (such as pain, and sleep disturbance), depressive symptoms, and unhealthy lifestyle choices (such as a poor diet and sedentary lifestyle) may function as mediation pathways that result in unregulated inflammation and depression. Stress, nutrition, depression, and adverse childhood experiences can all affect the gut microbiome and increase intestinal permeability, which is another route to elevated inflammatory responses [42]. Studies have shown that fruits, vegetables and edible legumes contain high levels of phytochemicals that have anti-inflammatory effects [90, 91]. Camu Camu (CC) has significant antioxidant and anti-inflammatory potential [92]. Treatment of high-fat/high-sucrose (HFHS)-fed mice that are treated with CC prevent weight gain, reduce fat accumulation, attenuate metabolic inflammation and endotoxemia, and mice treated with CC exhibit enhanced glucose tolerance as well as insulin sensitivity [93]. Consuming fruits and vegetables rich in polyphenols may improve cognitive and emotional health by reducing oxidative stress and inflammation [94]. To investigate the effect of the gut microbiota on stress-induced depressive behavior, Chevalier et al. adopted an unpredictable chronic mild stress (UCMS) mouse model of depression as well as fecal microbiota transfer (FMT) from stress donors to naïve mice [95]. Results revealed that microbiota transfer conveyed behavioral symptoms of depression and reduced adult neurogenesis in recipient mice [95]. In addition, the metabolomic analysis demonstrated that FMT mice underwent changed fatty acid metabolism characterized by a deficiency of lipid precursors of endocannabinoid (eCB), which led to the compromised activity of the eCB system of the brain [95]. The eCB system plays its pleiotropic role via multiple neuronal processes. eCB system regulates adult neurogenesis through CB1 receptors expressed by neural progenitor cells [96]. CB1-deficient mice exhibit damage neural progenitor cell proliferation, self-renewal as well as neurosphere production, while CB1 receptor agonists enhance neurogenesis [97, 98].

A randomised controlled trial study by Ren et al. showed that an almond-based low carbohydrate diet (LCD) improved depression and glucose metabolism in patients with type 2 diabetes by modulating gut microbiota and glucagon-like peptide-1 (GLP-1) [99]. Almond-based LCD significantly reversed depressive-like behaviour and glycosylated haemoglobin, while significantly increasing the number of short-chain fatty acid (SCFA)-producing bacteria Roseburia, Ruminococcus and Eubacterium. This suggests that the role of almond-based LCD in improving depression in type 2 diabetic patients may be related to its stimulation of the growth of SCFA-producing bacteria, increased SCFA production and activation of the free fatty acid 2 (FFA2) receptor (known as GPR43), and further maintenance of GLP-1 secretion.

Brain-gut axis plays important role in depression and obesity. The gut microbiota has been proven to interact with various organs, including the brain. Gut microbiota and its metabolites may act directly or indirectly on the brain via vagal stimulation to regulate metabolism, obesity, body homeostasis and energy balance as well as central appetite and food reward signals, which play a vital role in obesity [100]. There is a growing body of data suggesting that the gut microbiota coordinating multiple bodily functions is closely linked to the immune, metabolic and nervous systems and that dysbiosis of the gut dysbiosis disrupts the homeostasis between these systems [101]. The GBA is a bidirectional connection between the gut microbiota and the brain [100]. GBA influences physiological function and behavior through three different pathways (neural pathways, endocrine pathways, and immune pathways) [100, 102]. The neural pathway mainly consists of the enteric nervous system and the vagus nerve; the endocrine pathway mainly affects the neuroendocrine system of the brain, especially the HPA axis as well as the immune pathway [100, 102]. In particular, the signals from a brain influence the motor, sensory and secretory patterns of the gastro-intestinal tract, regulate inflammatory processes and influence the structure of the gut microbiota, and in turn, visceral information from gastro-intestinal features can affect brain function [103]. For example, neuroendocrine hormones (e.g., corticosterone) alter intestinal permeability, barrier function, and communicate with immune cells regarding cytokine secretion, and immune cells release cytokines important in the host responses to inflammation and infection [104]. Some alterations in the gut microbiota can modulate host metabolic pathways and dietary behavior through GBA, leading to obesity [105]. Recently, the role of the microbiota as an important factor in regulating gut-brain signaling has emerged and the concept of the microbiota-GBA has been established [105]. The GBA is proposed as the focus of new scientific and clinical research on its possible sites of systemic therapeutic interventions for depression and obesity [106].

### The potential role of neuroplasticity in depression and obesity

Neuroplasticity is characterized as the potential of the brain to undergo neurobiological changes in responding to external stimuli [107]. It is currently accepted that the mammalian brain shows continuous plasticity at all stages of life. The plasticity of neurons enables the CNS to learn newly acquired skills and to engage in ongoing learning and memory. In addition, neuronal plasticity allows the reorganization of neuronal networks in response to environmental stimuli as well as recovery after lesions [108]. Neuronal plasticity can be achieved through neurogenesis, apoptosis, synapse-dependent activity, as well as reorganization of neuronal networks [109]. Stress and depression are usually correlated, and studies have shown that chronic stressful events commonly accelerate depressive episodes in vulnerable individuals. Advanced brain functions are proposed to require synaptic frequency decoding, and changes in synaptic activation frequency may cause an increase or decrease in the long-term efficiency of these synapses, which may lead to long-term potentiation (LTP) or long-term depression (LTD) [110]. Hwang et al. showed that the intensity of synaptic plasticity (LTP and LTD) at Schaffer lateral branch-CA1 synapses in hippocampal isolated slices of obese male mice was lower compared to sex-specific controls [111]. This suggests that, like mice in the depression model, HFD-obese mice also affect hippocampal synaptic plasticity. In neuroscience research, depressive-like behaviors and chronic stress have been related to damage to neuroplasticity, like neuronal atrophy as well as synaptic loss in the medial prefrontal cortex (mPFC) and hippocampus [112]. Neuroplasticity as a downstream mechanism of the effect of novel fast-acting antidepressants such as ketamine has stimulated great interest in the mechanisms of neuroplasticity [113]. Among them, α-amino-3-hydroxy-5-methyl-4-isoxazolepropionic acid receptor (AMPAR) activation, BDNF and mammalian/mechanistic target of rapamycin (mTOR)-mediated signaling, synaptic protein expression and synaptogenesis are framed with a focus on neuroplasticity to explain the potent and sustained depression treatment effects of these compounds [113].

The hypothesis of neuroplasticity in depression suggests that downstream effects of antidepressants, such as increased neurogenesis, contribute to improved cognition and mood [21]. BDNF is a growth factor that modulates neurite growth, functional neuronal connections, synapse formation, as well as synaptic plasticity in the CNS [114]. BNDF signaling was proved to be required for the antidepressant effect of ketamine [115]. Activation of the high-affinity BDNF receptor, tropomyosin receptor kinase B (TrkB), is essential for antidepressant-related behaviors [114]. Numerous reports using depression-like behaviors or different animal models manipulating the expression of BDNF or its receptor TrkB suggest that BDNF/TrkB participates in the pathophysiology of depression as well as the mechanism of action of antidepressant treatments [114, 116]. Enhanced translation and/or release of BDNF and activation of the BDNF receptor target TrkB may lead to further activation of downstream pathways essential in synaptic plasticity. BDNF-mediated activation of the TrkB receptor activates the PI3K/Akt signaling pathway and downstream activation of the MEK-MAPK/Erk signaling pathway [115]. Both pathways promote protein translation by activating the mechanistic target of rapamycin complex 1 (mTORC1) [117]. The acute activation of mTOR and protein translation may trigger sustained changes in synaptic plasticity leading to long-term effects of ketamine. Preclinical studies have shown that sodium-glucose co-transporter 2 inhibitors (SGLT2i) improve cognitive dysfunction, reduce oxidative stress, and neuroinflammation, and improve neuronal plasticity and mitochondrial brain pathways in obese and type 2 diabetes mellitus (T2DM) mice [118]. The mTOR serves as a trophic sensor with critical homeostatic functions in modulating energy metabolism, supporting neuronal growth as well as plasticity [115]. In addition, SGLT2i restored mTOR to its activated physiological state and prevented the onset or progression of neurodegenerative diseases [118].

Neuroplasticity due to chronic sugar intake has been shown to reduce impulse control and thus resistance to high-fat/high-sugar foods, leading to an obesity epidemic [119]. Akhaphong et al. showed that placental mTORC1 is the mechanistic connection between placental function and the programming of obesity as well as insulin resistance in adult offspring [115]. A study by Li et al. found that 8 weeks of HFD-feeding was effective in inducing metabolic disorders, including obesity as well as hyperlipidemia, in mice. Interestingly, the mice also exhibited depressive and anxious behaviors [120]. Li et al. concluded that HFD-feeding inhibited AMPK phosphorylation and induced mTOR phosphorylation [120]. After 28 days of treatment with the mTOR inhibitor rapamycin, autophagy and BDNF levels were elevated [120]. This suggests that improvement of lipid metabolism or enhancement of autophagy via the AMPK/mTOR pathway may be a potential candidate target for the therapy of obesity and depression.

### The potential role of HPA axis dysregulation in depression and obesity

The HPA axis, which consists of the hypothalamus, pituitary and adrenal glands, modulates the production of glucocorticoids and is associated with the pathophysiology of psychiatric disorders [40]. During stress conditions, the HPA axis promotes transient physiological adaptations that usually resolve after the stress stimulus is not present. Both psychological and physiological stresses activate the HPA axis, which stimulates the release of corticotrophin-releasing factor (CRF) and arginine vasopressin (AVP) from the hypothalamic paraventricular nucleus (PVN), and the HPA axis is an essential component of the stress response system [40, 121]. AVP activates the locus ceruleus-norepinephrine neuromodulatory system, triggering a “fight or flight” response (regulated by the epinephrine and the norepinephrine), while CRH acting on the pituitary gland, which in response secretes adrenocorticotropic hormone (ACTH) into the bloodstream [40]. Once ACTH arrives in the adrenal glands, it initiates the release of cortisol or corticosterone, an anti-inflammatory hormone, and mediates the physiological behavioral response to stress [40]. In normal conditions, negative feedback from cortisol on CRH and ACTH ensures HPA homeostasis via the activation of glucocorticoid receptors (GRs) as well as mineralocorticoid receptors (MRs) [40]. CRF and AVP both induce the secretion of ACTH from the anterior pituitary gland, which increases the release of ACTH, causing an elevated level of circulating glucocorticoid (GC) and inhibiting the secretion of CRF and AVP from the hypothalamus, forming a reverse feedback circuit [122]. Chronic stress is known to overstimulate the HPA axis and the regulation of corticosterone secretion, which is associated with abdominal obesity [123, 124]. Studies have shown that increased physical activity can normalize corticosterone secretion and thus have a positive impact on physical and mental health. A randomized controlled trial by Lasselin et al. found that immune and behavioral responses to lipopolysaccharide (LPS) differed little between obese and normal-weight subjects, but that cortisol responses to LPS were significantly attenuated in obese individuals and that higher body fat percentage was associated with lower cortisol responses to LPS [125]. This suggests that young and healthy obese individuals do not have increased behavioral sensitivity to cytokines, but have diminished cortisol responses to immune challenge.

Over the last decades, several hypothalamic axis abnormalities associated with stress overreaction have been identified in patients with depression. These include alterations such as excessive CRF secretion in the paraventricular nucleus of the hypothalamus, impairment of negative feedback in the HPA axis, enlarged adrenal glands, hypercortisolism, and reduced inhibition of cortisol by dexamethasone [126]. Neuroendocrine studies have demonstrated HPA axis hyperactivity in patients with MDD. Changes in the HPA axis in depressed patients have been consistently reported, with approximately 30% of depressed patients having higher cortisol levels [127]. It has been shown that over 40-60% of depressed patients experience hypercortisolemia. Increased stress-induced GC secretion may enhance mood and motivation [128]. Microbiota-induced hyperactivity of the HPA axis and inflammation have also been implicated in causing depression [129]. Pro-inflammatory cytokines exert the potential to activate the HPA axis, and patients with depression also have a hyperactive HPA axis. Furthermore, HPA axis dysfunction decreases BDNF expression, inhibits 5-HT synthesis, reduces Glu receptor expression, and even disrupts neuroplasticity as well as neural circuits [122, 130]. Related studies suggest that an excess of pro-inflammatory cytokines inhibits the negative feedback of the HPA axis, growing the permeability of the BBB, reducing 5-HT synthesis, interfering with the glutamatergic system, and ultimately leading to depression [131, 132]. More than half of depressed patients display negative feedback dysfunction of the HPA axis, including chronic increases in circulating GC and ACTH [133]. Chronic stress and stress hormones such as glucocorticoids trigger metabolic changes, including obesity and diabetes.

A growing body of research suggests that chronic stress-related stimulation of the HPA axis and the causing excess glucocorticoid exposure may exert a fundamental role in the development of visceral obesity [134]. FGF21 is a longevity factor that coordinates the interaction between energy metabolism and stress response [135, 136]. FGF21 is a general stress factor that not only alters energy metabolism but also activates multiple rescue processes directly or indirectly by stimulating the secretion of lipocalin and hormones in the HPA axis through the AMPK signaling pathway and several other pathways [137]. Recent studies have shown that FGF21 therapy can relieve many metabolic diseases, such as obesity, type 2 diabetes as well as some cardiovascular diseases. FGF21 can control stress response and metabolism by regulating the function of the somatotropic axis and HPA pathway [135]. Werdermann et al. showed that obese animals show an overactive HPA axis, leading to adrenal hyperplasia [137].

### The potential role of the neural cell nucleus and neural circuit in depression and obesity

Although neuroplasticity is important for depression and obesity co-morbidity, there is growing evidence that it is significant to explore the neural mechanisms of depression and obesity co-morbidity at the level of the neural cell nucleus as well as the neural circuit [138]. Xia et al. suggest that melanocortin 4 receptor (MC4R) neurons, located in the bed nucleus of the stria terminus (dBNST), are involved in psychiatric-related body weight regulation by receiving α5-containing GABA_A_ receptor and serotonergic GABAergic projections from hypothalamic AgRP neurons [138]. Overall, targeting GABA_A_R-α5 and 5HT_3_R within MC4R^dBNST^ neurons contributed to rescuing HFD-induced anxiety and depression, thereby reducing body weight by simultaneously reducing HFD cravings and enhancing a healthy low-fat diet in mice [138]. In addition, studies have shown that mixtures of zonisamide-granisetron cocktail restore mental normalcy and promote weight loss by targeting the GABA_A_R-α5 and 5HT_3_R pathways, respectively, by altering food preferences toward a healthy low-fat diet [138].

Recent studies by Hallihan et al. have found that affective neural circuits and inflammatory markers are associated with symptoms of depression and anxiety in comorbidities of obesity [139]. They used the current feasibility study using functional neuroimaging and biospecimen data to identify whether changes in inflammatory markers, fecal short-chain fatty acids, as well as neural circuit-based targets predicted depression and anxiety outcomes in comorbid obese participants, and preliminary correlation analyses showed significant correlated changes in three inflammatory markers (IL-1RA, IL-6, and TNF-α) and five neural targets (negative influence circuit, positive influence circuit, and default mode circuit) at 2 months [139]. Further, they found that changes in IL-1RA and TNF-α at 2 months, as well as changes in three neural targets (negative influence circuit and positive influence circuit), correlated with changes in depression and anxiety symptoms at 6 months [139]. Perinatal exposure to maternal obesity, metabolic disorders (including diabetes and hypertension), and unhealthy maternal diets have long-term effects on the behavior and physiology of offspring [123]. Evidence from epidemiological studies suggests that maternal obesity and metabolic comorbidities increase the risk of attention deficit hyperactivity disorder (ADHD), autism spectrum disorder (ASD), depression, schizophrenia, eating disorders (food addiction, anorexia nervosa, as well as bulimia nervosa), and cognitive impairment in offspring [140]. In addition, during pregnancy, inflammation in the offspring’s brain impairs the development of neural pathways critical to behavioral regulation, such as the serotonergic, dopaminergic, and melanocortinergic systems [123].

### The potential role of genetic association in depression and obesity

Mendelian randomisation (MR) studies have shown that obesity and the development of depression are causally related [141]. To provide a better comprehension of the relationship between obesity and depression, Speed et al. conducted a MR study of the relationship between adiposity, non-adiposity, height and depression, using results from the UK Biobank (n = 332,000) and the Psychiatric Genomics Consortium (n = 480,000) genome-wide association studies [141]. The results of the study found that both adiposity and height (short stature) were causal risk factors for depression, whereas non-adiposity was not [141]. In addition, they note that reducing fat mass will reduce the risk of depression. O’Loughlin et al. performed a two-sample MR using genetic summary statistics (15771 cases and 178777 controls) from the recent Genome-Wide Association Study (GWAS) of depression in East Asian ancestry. i.e., single nucleotide polymorphisms (SNPs) associated with BMI and SNPs associated with waist height ratio (WHR) were selected as genetic instrumental variables and inverse variance weighting (IVW) method to estimate the causal relationship between BMI and WHR and depression [142]. This GWAS data provides the first MR evidence that high obesity is associated with a lower risk of depression in a population of East Asian ancestry living in East Asia. Liao et al. performed genome-wide genotyping of 106,604 unrelated individuals from a Taiwanese biobank to derive polygenic risk scores for BMI and MDD to assess their effects on obesity-associated traits [143]. This study showed that MDD polygenic risk score (PRS) was positively associated with waistline, hipline, waist-hip ratio, body fat percentage, BMI, overweight (BMI ≥ 25), and obesity (BMI ≥ 30) [143]. The results of the meta-analysis of interactions suggest a genetic mechanism for the increased risk of obesity in patients with depression [144]. Rivera et al. showed that depression enhanced the effect of FTO variants on BMI, with a 2.2 increase in BMI per rs9939609 risk allele (A) in depressed patients compared to mentally healthy controls [144]. This suggests that alterations in key biological processes associated with depression may interact with FTO risk alleles to increase BMI or obesity risk.

In a cross-trait meta-analysis, Amare et al. identified 14 genetic loci (including NEGR1, CADM2, PMAIP1, and PARK2) linked to obesity and response to treatment with Selective serotonin reuptake inhibitors (SSRIs) [145]. Weight gain is a side effect of antidepressants and antipsychotics that can cause many comorbidities and reduce life expectancy [146]. The majority of included studies demonstrated a 5% weight gain in individuals treated with antidepressants; however, Quetiapine, Haloperidol, Trifluoperazine, Risperidone, Aripiprazole, Olanzapine, and Clozapine resulted in≥7% weight gain from baseline [146]. Some antidepressants, such as Mirtazapine, show significant levels of weight gain, while others, such as Bupropion, show weight loss effects [147]. Therefore, controlling undesired weight effects is an important consideration in the selection of antidepressants.

## Major adipocytokines and lipokines as well as current relevance to obesity and depression

Studies have established that adipose tissue is a dynamic organ that performs several vital physiological processes. It consists of many cell types: e.g., adipocytes, pericytes, preadipocytes, vascular endothelial cells, macrophages and fibroblasts [148]. However, the major cells present in the adipose tissue are the mature adipocytes. Three types of adipocytes are found in mammals and are usually classified according to their color appearance: white, brown and beige/brite/brown adipocytes [149, 150]. White adipocytes are primarily involved in energy storage and mobilization, while brown adipocytes are chiefly involved in nonshivering thermogenesis [149]. Adipose tissue and adipocytes store the energy, produce adipokines and lipokines to regulate the energy supply and are crucial players in the endocrine system. Adipokines and lipokines act on the target tissues to regulate energy supply, lipid metabolism and immune response [151]. Adipokines are derived mainly from adipocytes, even though some members are synthesized by other types of cells as well [152]. These adipocytokines resemble classical cytokines and have pleiotropic functions that influence biological processes throughout the entire organism [152]. It has long been suggested that adipocytokines have a profound protective role in the pathogenesis of diabetes and cardiovascular disease [153]. In recent years, it has been found that adipocytokines also act as an effective modulator of depressive and anxiety-like states, but the mechanism of action of adipocytokines in modulating depressive and anxiety-like behavior is still unclear. Recently, more and more adipocytokines have been recognized, such as leptin, adiponectin, plasminogen Activator I (PAI-1)/visfatin, MCP-1, RBP4, pre-B-cell colony enhancing factor, resistin, apelin, chemerin, aprotinin, vaspin, and others [152]. Leptin and adiponectin are by far the most thoroughly studied adipocytokines. Numerous studies have demonstrated that leptin and adiponectin regulate brain cell proliferation, survival as well as synaptic plasticity via regulation of cellular metabolism and suppression of inflammatory responses [154, 155]. Since leptin and adiponectin play an essential role in brain diseases, these two adipocytokines have been described in detail. In addition, in Table 1, we summarize the representative adipocytokines and their current relevance to obesity and depression.

**Table 1:** Major adipocytokines and current relevance to obesity and depression.

**NO.**	**Major adipocytokine**	**Source**	**Functions**	**Relevant to obesity**	**Relevant to depression**	**References**
1	Leptin	white adipocytes, brown adipocytes	regulate energy balance, body weight, metabolism and endocrine function	reduced appetite; elevated energy expenditure; decreased heat loss; reduced insulin secretion, increased by fat mass	leptin levels are higher in patients with moderate to severe depression than in those with mild depression	[20, 162, 168]
2	Adiponectin	mature white adipocytes, brown adipocytes	enhance insulin sensitivity, decreased inflammation	decreased by obesity, increased insulin sensitivity, decreased gluconeogenesis, increased fatty acid catabolism,	adiponectin works through AdipoR1 receptors on 5-HT neurons to mediate depression-related behaviors in a sex-dependent manner, decreased by oxidative stress, decreased by endoplasmic reticulum stress	[10, 169]
3	PAI-1	hepatocytes, endothelial cells, platelets, adipocytes, and cardiac myocytes (*in vitro*)	the main physiological inhibitor of plasminogen activators/plasmin system; 50-kilodalton glycoprotein of the serine protease inhibitor family; inhibition of the tissue- and urokinase-type plasminogen activator	PAI-1 is frequently elevated in obesity	elevated/ decreased plasma PAI-1 levels in depressed patients; PAI-1 knockout mice are a model of resistance to antidepressants such as SSRIs	[18, 170–173]
4	MCP-1	endothelium, fibroblasts, macrophages, monocytes, vascular endothelial cells, smooth muscle cells, astrocytes and microglia	strong monocyte chemotactic active molecule	MCP-1 plays an essential role in obesity-associated monocyte/macrophage infiltration	decreased serum MCP-1 levels in MDD patients	[174, 175]
5	RBP4	white and brown adipocytes, liver, adipose tissue	RBP4 is a protein in the lipocalin family and a specific carrier protein of vitamin A in the blood	RBP4 related to systemic insulin resistance, dyslipidemia and obesity	serum RBP4 levels were substantially lower in MDD patients than in controls	[11, 176]
6	PBEF/ visfatin	high levels in visceral fat, bone marrow, liver tissue and muscle cells, but also a variety of other tissues, including the placenta, kidney, heart and lung.	a pro-inflammatory cytokine that has functions related to cellular metabolism, inflammation and immune regulation.		visfatin reduces apoptosis and necrotic cell death in the CA1 region of the hippocampus of ischemia/reperfusion stroke rats, contributing to a neuroprotective effect	[177–181]
7	Resistin	white and brown adipocytes, macrophages	presents as trimer/hexamer in plasma, and targets specific receptors TLR4 or Adenylyl CAP1, triggering various intracellular signal transduction pathways to induce vascular inflammation, lipid accumulation, and plaque vulnerability	in rodents, resistin is increased in high-fat/high-carbohydrate-fed, obese states	lower serum resistin levels in MDD patients compared to healthy controls	[182–184]
8	Apelin (APLN)	adipose tissue also secreted from various tissues in the cardiovascular, digestive, urinary, and CNS	follicle development, regulating glucose and lipid metabolism, modulating insulin secretion, cardiovascular function, blood pressure, angiogenesis, drinking behavior	mediate glucose and lipid metabolism, regulate insulin secretion, plasma apelin concentrations are increased in obesity	Apelin-13 reverses depression-like behavior in CSDS rats model, decreased depressive behavior in sucrose preference and tail suspension tests	[185–188]
9	Chemerin	white adipose tissue	regulate adipogenesis, insulin sensitivity, and immune response	systemic levels of chemerin are increased in obesity	decreased depressive behavior in forced swim and tail suspension tests, better learning and memory functions	[189–191]
10	lipid chaperone FABP4	highly expressed in white adipocytes, brown adipocytes, macrophages, endothelial cells,	lipid chaperone protein, maintain glucose homeostasis and facilitate communication between energy storage systems and distant organs	long-term involvement of FABP4 in obesity under conditions of immunometabolic stress, regulate metabolic and inflammatory pathways in response to fatty acids	potential role in cell signaling, neuronal development and synaptic function	[192, 193]
11	IL-6	white and brown adipocytes, macrophage, endothelial cells, immune cells	IL-6 is produced rapidly and transiently in response to infection and tissue injury and promotes host defence by stimulating the acute phase response, hematopoiesis and immune response	acutely elevated IL-6 levels aid in fasting or exercise-induced fat mobilization, IL-6-dependent induction of leptin and free fatty acid release from adipocytes	patients with MDD have high levels of various pro-inflammatory cytokines, such as IL-6	[194–196]
12	Nrg4	activated thermogenic adipose tissue, brown adipocytes, hepatocytes	modulate glucose and lipid metabolism and energy balance	Nrg4 is substantially down-regulated in mouse and human obesity	play a role in neural development and function, Nrg4 is a major novel regulator of dendritic arborizations in the developing cerebral cortex	[197, 198]
13	Omentin	omental adipose tissue, macrophage, endothelial cells, the stromal-vascular fraction of visceral adipose tissue	regulate insulin sensitivity, alter inflammatory states	prolonged insulin-glucose infusion in healthy individuals induces a significantly reduced plasma omentin-1 concentration, serum omentin-1 concentrations were significantly lower in overweight and obese subjects than in lean individuals	omentin protects against the decrease in cell viability induced by the pro-inflammatory cytokine TNF-α, omentin promotes the growth and survival of NSCs *in vitro* by activating the Akt signaling pathway	[199, 200]
14	Adipsin	white adipocytes, brown adipocytes	reduced inflammation by chemotaxis, decreased inflammation by clearance of dead cells	decreased by insulin, adipsin serum concentrations are strongly related to obesity, adipsin is downregulated in obesity	mood disorders have lower adipsin levels	[8, 201, 202]
15	Vaspin	white and brown adipocytes, preadipocytes, visceral and subcutaneous adipose tissues	play a crucial role in osteogenesis, steroidogenesis, the formation of blood vessels, and food intake, vaspin action on cell apoptosis and proliferation, serine protease inhibitor, improves hyperglycemia	vaspin levels are higher in obese subjects, vaspin mRNA expression was increased in human adipose tissue, vaspin may be a target for the treatment of insulin resistance and inflammation associated with obesity, decreased food intake	elevated vaspin serum concentrations are associated with impaired health levels and leptin serum concentrations	[9, 199, 203, 204]

Note: AdipoR1, adiponectin receptor 1; 5-HT, 5-hydroxytryptamine or serotonin; PAI-1, Plasminogen activator inhibitor type 1; SSRIs, selective serotonin reuptake inhibitors; MCP-1, Monocyte chemoattractant protein-1; MDD, major depressive disorder; RBP4, Retinol Binding Protein 4; PBEF, Pre-B cell colony enhancing factor; TLR4, Toll-Like Receptor 4; CAP1, Cyclase-Associated Protein 1; APLN, Apelin; CNS, central nervous system; CSDS, chronic social defeat stress; FABP4, fatty acid-binding protein 4; IL-6, Interleukin 6; Nrg4, Neuregulin 4; TNF-α, tumor necrosis factor-alpha; NSCs, neural stem cells; AKT, serine-threonine protein kinase.

In the past, studies on the adipose secretome have mainly focused on polypeptide adipokines. Adipose-derived blood-borne lipids (“lipokines”) are a distinct endocrine factor that has been relatively hotly studied in recent years [156]. Lipokines are fatty acids that influence lipid metabolism and behave like hormones [157]. Interestingly, one of the systemic areas with active lipid metabolism in adipose tissue, which releases diverse lipids into the bloodstream to interact with distant organs [156]. Lipids are also closely related to intracellular fatty acid metabolic pathways and can transfer the intracellular energy of adipocytes to other non-adipose tissues. Unexpectedly, alterations in lipid metabolism might also be connected to the emergence of mental disorders like depression. It has been proposed that increased adipose tissue is linked to chronic inflammation and pro-inflammatory factors that inhibit lipokines production and that chronic inflammation associated with visceral obesity inhibits lipockines production and perpetuates inflammation [158]. There is no doubt that inflammatory dysregulation plays an instrumental role in the pathogenesis of depression, and lipokines may also influence depression via this pathway. Lipokines, mainly including lysophosphatidic acid (LPA) and monounsaturated palmitoleic acid. LPA can interact with the central nervous system and also have endocrine impacts on systemic tissues. In the serum, palmitoleic acid is one of the most prevalent fatty acids. Its circulating levels change according to metabolic conditions. Besides, 12,13-dihydroxy-9Z-octadecenoic acid (12,13-diHOME) is the best-studied lipokines secreted by brown fat, produced by the dihydroxylation of linoleic acid. FAHFAs (fatty acid esters of hydroxy fatty acids) are also lipokine. FAHFAs are secreted by adipocytes into plasma, and in the organism, FAHFA levels in adipose tissue and plasma are associated with insulin sensitivity and a decline in insulin-resistant states. In Table 2, we summarize the representative lipokines and their current relevance to obesity and depression.

**Table 2:** Major lipokines and current relevance to obesity and depression.

**NO.**	**Major lipokines**	**Source**	**Functions**	**Relevant to obesity**	**Relevant to depression**	**References**
1	Palmitoleic acid (Palmitoleate)	white adipocytes	decreased inflammation, decreased atherogenesis, increased glucose homeostasis, enhances whole-body insulin sensitivity	PLA (16:1n-7) has hormone-like properties and improves several metabolic parameters that are damaged in obesity	based on fatty acid analysis, the palmitoleic acid was remarkably altered in mice with depressive-like behavior.	[205–207]
2	DNL FAHFAs	white adipocytes	increased by glucose metabolism, increased by DNL, increased glucose tolerance, increased insulin secretion	dysregulation of DNL is often observed in a variety of metabolic abnormalities, including obesity	expression of de novo fatty acid was enhanced in HFD mice with depression-like behavior	[8, 207, 208]
3	12,13-diHOME	brown adipocytes	increased by cold exposure, increased by exercise, increased skeletal muscle fatty acid oxidation, increased fatty acid transport	increasing 12,13- diHOME levels may prevent and treat obesity and metabolic diseases	unknown	[8, 209]
4	LPA	LPA is found in virtually all biological fluids	influences diverse cellular and organismal processes, including proliferation and growth, survival, development, chemotaxis, vasoregulation, and calcium dynamics	ATX-LPA-LPA1-6 signaling axis in the development of metabolic disorders, including obesity, insulin resistance, as well as damaged glucose homeostasis	LPA management improves depression and anxiety, LPA treatment may regulate the activation of microglia, which plays a critical role in psychiatric disorders such as depression	[210, 211]

Note: DNL, de novo lipogenesis; FAHFAs, fatty acid esters of hydroxy fatty acids; 12,13-diHOME, 12,13-dihydroxy-(9Z)-octadecenoic acid; LPA, lysophosphatidic acid; HFD, high-fat diet; ATX, autotaxin.

### The biology of leptin and its relationship to obesity and depression

To date, leptin is by far the finest instance of a successfully moving from discovery to clinical application [9]. The role of leptin in neuroendocrine has been extensively studied. In 1994, Friedman’s team cloned for the first time a hormone-like substance, leptin, secreted by adipocytes and found that it has appetite control and weight reduction effects [159]. The endocrine hormone leptin exerts a pivotal role in modulating food intake and body weight through the action of the hypothalamus [160]. Leptin, a key adipose-derived hormone that regulates eating behavior and body weight, is associated not only with obesity but also with depression. Intraperitoneal injection of leptin into C57BL/6J mice reduced depressive-like behavior in the forced swimming test as well as the tail suspension test [161]. Relevant studies have suggested that leptin levels are higher in patients with moderate to severe depression than in those with mild depression or mild to no depression, and that body mass index (BMI) is higher in patients with moderate to severe depression than in those with mild or mild depression [20]. And after adjusting for multiple factors such as age, gender, and race, leptin levels remained a key predictor of depression [162]. This suggests that leptin may mediate the progression of depression in obese individuals or be a common mechanism causing depression and obesity.

Studies have indicated that the neuroprotective effects of leptin may be related to the leptin/JAK2/STAT3/PGC-1signaling pathway or the leptin-mediated PI3K/Akt/mTOR signaling pathway [163, 164]. Obesity damages leptin-induced regulation of BDNF expression and synaptogenesis, which is thought to be related to the onset of depression. Ginsenoside Rb1 is a major bioactive ingredient of ginseng, and Wu et al. showed that chronic treatment with ginsenoside Rb1 improved central leptin sensitivity, the leptin-JAK2 -STAT3 signaling pathway, as well as the regulation of leptin-induced BDNF expression in the prefrontal cortex of obese mice induced by a HFD [165]. This suggests that supplementation of Rb1 may be a useful way to treat obesity-related psychiatric disorders. Leptin has been proven to affect hippocampal synaptic plasticity [166, 167]. Synaptic plasticity is the most functionally critical form of neuroplasticity, and it exerts an essential role in the neuropathogenesis of several psychiatric disorders. Leptin regulates the efficacy of hippocampal transmission synapses, including LTP and LTD [20]. LTP and LTD are two types of mechanisms that affect impaired cognitive and emotional function in MDD [167]. Under intense, sustained stimulation, increased neuronal firing enhances LTP by strengthening synapses, and LTD causes an activity-dependent decrease in neuronal synaptic efficacy and connectivity [167]. Leptin receptors are widely expressed in various regions of the brain, particularly the hippocampus, which is a crucial region for learning and memory formation as well as emotion regulation. Leptin can cross the BBB and bind to a specific leptin receptor (LepRb) [212]. LepRb deficiency also resulted in memory and cognitive impairment with altered hippocampal synaptic plasticity [213]. It was shown that especially in hippocampal structures, CA1/CA3 regions and dentate gyrus (DG) widely express LepRb mRNA. Several pieces of evidence suggest that leptin is a potent mediator of excitatory synaptic transmission at hippocampal CA1 synapses [213]. The binding of leptin to LepRb isoforms associated with the JAK2-STAT3 signaling pathway causes induction of SOCS3, which terminates JAK2 activity via ubiquitin-mediated degradation of JAK2, thereby terminating the leptin signaling pathway. Enhancing or sustaining the activation of SOCS3, increases obesity-induced leptin concentrations in the blood, leading to leptin resistance. Liu et al. showed that LepRb knockdown-induced depression-like behavior was correlated with STAT3/SOCS3 signaling pathway [19]. LepRb may serve as a novel direction for depression treatment in the future.

### The biology of adiponectin and its relationship to obesity and depression

To date, adiponectin is one of the most extensively studied adipokines [214]. Adiponectin, also called 30-kDa adipocyte complement-associated protein (Acrp30), is a type of hormone secreted by adipocytes [215]. The primary role of adiponectin in arcuate nucleus promelanocortin (POMC)-expressing neurons is excitatory, depolarizing neurons, reducing inhibitory synaptic inputs, as well as increasing their responsiveness [216]. The current study suggests that adiponectin directly mediates the cellular activity of arcuate POMC and neuropeptide Y/Agouti-related peptide (NPY/AgRP) neurons [216]. Adiponectin inhibits orexigenic NPY neurons under hypoglycemic conditions and activates orexigenic POMC neurons, thereby attenuating appetite and food intake under fasting or hypoglycemic conditions [166]. In cases of obesity, insulin resistance and type 2 diabetes, blood levels of adiponectin are reduced [215]. Moreover, intracerebroventricular injection of adiponectin -neutralizing antibodies can induce stressful depressive behavior [217]. Adiponectin receptors (AdipoRs) are widely present in the rodent brain, including the hypothalamus, brainstem, prefrontal cortex, as well as hippocampus [155, 217, 218]. While AdipoR1 was highly expressed in the skeletal muscle, AdipoR2 was strongly expressed in the liver [215]. AdipoRs are located on neurons that participated in metabolic regulation in the hypothalamus, including arcuate POMC and NPY/AgRP neurons [216]. Studies have shown that AdipoRs expression is regulated by adiponectin and dopamine signaling pathways [219]. Adiponectin can initiate Notch signaling in the hippocampus by upregulating ADAM10 and Notch1, which are two pivotal molecules in Notch signaling [220]. It has been shown that the removal of AdipoR1 from dopamine neurons could enhance neuronal and anxiogenic responses to suppress stress [219]. The effects of AdipoR1 on neuronal activity and behavior were found to be abolished in dopamine-neuron deficient AdipoR1 mice with VTA infusion of lipocalin [219]. This finding suggests that adiponectin regulates VTA dopamine neuron activity and that AdipoR1 is essential for adipokine-induced inhibition of dopamine neurons directly. Activation of AdipoR1 and AdipoR2 stimulates the activity of AMPK and p38 mitogen-activated protein kinase (p38MAPK), and recently, the p38MAPK signaling pathway was found to regulate adiponectin-induced phosphorylation of the glycogen synthase kinase 3β (GSK-3β) Ser389 inhibitory site [221]. A large body of data suggests that active GSK-3β is associated with increased susceptibility to mood disorders. And inhibition of GSK-3β may be linked to the treatment effects of antidepressants [222]. Thus, adiponectin-induced GSK-3β inhibition may be the basis for the mechanism of antidepressant action of adiponectin. Like LepRs, AdipoRs activate several overlapping signaling cascades, including Janus kinase 2/signal transducer and activator of transcription 3 (JAK2/STAT3), phosphatidylinositol-3-kinase (PI3K), insulin receptor substrate 1/2 (IRS1/2), forkhead box protein O1 (FOXO1) and AMP-activated protein kinase (AMPK) [216, 223]. So far, studies of adiponectin receptors have primarily focused on most aspects of the AMPK signaling pathway regulating metabolism in peripheral tissues [224]. AdipoRs exert a crucial role in the regulation of glucose and fatty acid metabolism via initiating a few signaling cascades that overlap with LepRs [216]. Adiponectin and its adiponectin -receptor interactions are complex processes. In the future, the receptor subtypes and neuronal circuits responsible for the antidepressant-like effects of adiponectin will need to be identified. It was shown that adiponectin expression was reduced in adipose tissue and blood of obese mice, and circulating adiponectin levels were also decreased in obese patients, however, the expression of adiponectin receptors was increased [225–227]. Liu et al. showed that in a chronic social defeat stress (CSDS) model of depression, plasma levels of adiponectin were reduced, which was associated with a reduction in the duration of social interaction [217]. They suggest that reduced adiponectin levels lead to elevated susceptibility to social aversion, pleasure deficit and learned helplessness, as well as to impaired glucocorticoid-mediated negative feedback in the HPA axis [217]. In addition, adiponectin activates neurogenesis in the hippocampus, which may facilitate its antidepressant-like behavioral effects [221].

### The biology of palmitoleic acid and its relationship to obesity and depression

Excessive fat accumulation in the body causes adipose tissue dysfunction. This impairment potentially results in elevated release and concentration of circulating free fatty acids, glycerol, hormones, as well as inflammatory cytokines [228]. All these modifications are linked to distinct health problems, such as dyslipidemia, hypertension as well as insulin resistance, collectively known as the “metabolic syndrome” [228]. The ratio of saturated fatty acids to monounsaturated fatty acids is essential in regulating biological membrane fluidity [229]. An imbalance in the ratio of these two may facilitate the development of several diseases, such as diabetes, and cardiovascular disease [229]. Stearoyl-CoA desaturase (SCD) (Δ9 desaturase) and hexadecenoic fatty acids (16:1) are the major monounsaturated fatty acids present in cells and tissues [230, 231] Recently, monounsaturated hexadecenoic fatty acids have been becoming considered biomarkers of health and have vital functions in physiology and pathophysiology [231]. In recent years, palmitoleic acid (cis-9-hexadecenoic acid, 16:1n-7) and its positional isomers 16:1n-9 and 16:1n-10 have gained much attention for their anti-inflammatory properties [231]. PLA (16:1n-7) is the most abundant member of the monounsaturated fatty acids family and the best studied. PLA (16:1n-7) was synthesized from palmitic acid in the presence of SCD-1 and fatty acid desaturase-2 [231]. It can be ingested via the diet and synthesized endogenously from other fatty acids, carbohydrates as well as amino acids [232]. PLA (16:1n-7) is often described as a lipokine capable of regulating a variety of metabolic processes such as increasing insulin sensitivity in muscle, prevention of endoplasmic reticulum stress, β-cell proliferation, and lipogenic activity in white adipocytes [157]. Cao et al. showed that the release of this fatty acid from adipose tissue inhibited steatosis in the liver and modified insulin signaling in muscle [157]. Collectively, this study showed that PLA (16:1n-7) acts as an anti-inflammatory agent in the adipose tissue of mice and contributes to reducing the effects of obesity. This shows that PLA (16:1n-7) inhibits adipocyte cytokine expression, while palmitic acid does not inhibit adipocyte cytokine expression, suggesting that adipocytes are the primary target of PLA (16:1n-7) [157]. Lopes A et al. showed that PLA (16:1n-7) improves systemic insulin sensitivity and glucose uptake into adipose tissue by regulating GLUT-4 and AMPK phosphorylation in HFD-fed mice [233]. Another study also discovered that PLA (16:1n-7) treatment may prevent the enhancement of transcription factors CEBPα and PPARγ in subcutaneous adipocytes in the inguinal groin of HFD-treated mice [234]. PLA (16:1n-7), described as a lipotropic hormone, may play diverse roles according to the organ and disease model under study [207]. According to fatty acid analysis, palmitoleic acid content was significantly altered in mice with depressive-like behavior. Moreover, the expression of acetyl coenzyme a carboxylase (ACC), SCD1, and fatty acid desaturase 1 and 2 (FADS1 and FADS2), which are involved in the fatty acid synthesis, fatty acid desaturation, and arachidonic acid synthesis, was enhanced in HFD mice with depressive-like behavior [207]. Thus, it is hypothesized that HFD-induced disturbances in lipid metabolism speed up the development of depression-like behaviors. Although beneficial effects of PLA have been observed in both *in vivo* and *in vitro* studies, there have not been sufficient human intervention studies to fully comprehend the physiological effects of palmitoleic acid [206]. Hence, more human-based studies are required to determine whether PLA has promising therapeutic potential.

### The biology of lysophosphatidic acid and its relationship to obesity and depression

LPA, also called monoacyl-sn-glycero-3-phosphate, is a lysophospholipid with a glycerol phosphate head group and a fatty acid moiety. It is not a single chemical entity, but represents a class of biomolecules with different fatty acid chain lengths and saturations [235]. Autotaxin (ATX) is a secreted enzyme that hydrolyzes lysophosphatidylcholine to generate LPA. A growing number of studies suggest that the ATX-LPA axis is involved in obesity and its associated metabolic complications [236, 237]. LPA is a lipid mediator which is produced by adipocytes through specific G-protein-coupled receptors and its synthesis is regulated in obesity. Rancoule et al. showed that reduced adipocyte LPA production was correlated with improved glucose tolerance in HFD-fed obese mice [238]. This suggests that LPA harms glucose homeostasis.

Synaptic signaling is a plastic dynamic process crucial for information processing at the level of brain cells and neuronal networks and is critical for regulating neuronal excitability as well as brain information processing [239]. The LPA-triggered signaling pathway induces rapid and reversible inhibition of excitatory and inhibitory postsynaptic currents. In excitatory synapses, the LPA1/Gαi/o protein/phospholipase C/myosin light chain kinase cascade acts at presynaptic sites [239]. LPA can act as potential partial messengers, modulating synaptic strength to accommodate the prior activity of neurons [239]. LPA primarily mediates its action by activating six known G protein-coupled receptors (GPCRs), and the protein products are named LPA1 to LPA6 [240]. LPA is activating six LPA receptors (LPAR1-6) and modulates different cellular activities, i.e. cell proliferation, cytoprotection as well as wound healing. LPA receptors are classically seven-transmembrane GPCRs activating heterotrimeric G proteins to signal transduction within the cell [235]. It is well documented that LPA and LPA receptor signaling pathways are required for the formation of mature synaptic connections, particularly glutamatergic synapses [241]. As one of the six characteristic G protein-coupled receptors (LPA1-6), the LPA1 receptor through which lysophosphatidic acid serves as an intracellular signaling molecule. Studies have shown that the deletion of LPA1 receptors causes anxiety as well as several behavioral and neurobiological changes that are closely related to depression [242]. Endogenous LPA signaling mediates activity-dependent inhibition primarily through LPA1 in an experimental model of synaptic plasticity [239].

In the hippocampus, genetic deletion of *Lpar1* leads to more immature dendritic spines in CA1 pyramidal cells and reduces matrix metalloproteinase 9 (MMP-9), which has been proven to be engaged in regulating synaptic plasticity [243]. In zebrafish *in vivo* experiments, the *Lpar3* gene exerts novel roles in regulating behaviors such as anxiety, social interactions, circadian rhythmic motor activity, and memory retention [244]. Gintonin, an exogenous LPA receptor ligand, was isolated from P. ginseng. Kim et al. suggested that gintonin-enriched fraction may be associated with the relief of depression-related symptoms induced by ginseng extract [245]. All this evidence suggests that LPA plays an essential role in both obesity and depression.

## Potential and future treatment of depression and obesity

The multifaceted effects of adipokines and lipokines on neurological and brain health and the dysregulation of adipokine and lipokine secretion may contribute to the co-morbidity of obesity and depression [166]. Exploring the pathogenesis of obesity and depression from the perspective of multiple adipokines and lipokines has beneficial implications for the future treatment of obesity and depression. Adipokines and lipokines such as leptin, adiponectin, PLA, and LPA may be critical targets for the future treatment of obesity and depression. Clinical studies have shown that individuals with POMC or LepRb deficiency typically experience severe obesity, bulimia and comorbidities, which can severely impact the patient’s quality of life and depressive behavior [246].

Diet and obesity have been proven to have a direct effect on mood, and stress-related mental diseases might result in alterations in eating patterns that affect weight [247]. Recent studies suggest that dietary interventions and energy restriction may help prevent depression and anxiety, which would serve as complementary therapies [248]. Ganoderma lucidum is a medicinal mushroom commonly used to improve quality of life, promote health, as well as enhance vitality. Studies have shown that ethanolic extract of Ganoderma lucidum ethanol extract (EEGL) has significant effects on feeding behavioral parameters, depression-like symptoms and locomotor activity in Swiss mice, as reflected by a significant decrease in body weight gain and food intake, a dose-dependent increase in water intake, and a decrease in immobility time in the forced swim test (FST) and the tail suspension test (TST) in Swiss mice [249]. These findings suggest that EEGL can reduce body weight gain and produce antidepressant-like effects.

Unhealthy eating patterns might be connected with an increased risk of depression or anxiety, while healthy eating patterns might reduce that risk. The first human study to show a link between diet and hippocampus volume mirrored findings from previous preclinical investigations in animal models, in which low nutritional food intake and excessive unhealthy food intake were independently linked with decreased left hippocampal volume, respectively [250]. Recent clinical studies have shown that diet can influence the physiological and immune functions of the body and has potential therapeutic strategies. The study found that 83% of participants who adhered to a ketogenic diet experienced significant reductions in fat mass and nearly 50% decreases in self-reported fatigue and depression scores over the study period [251].

Functional brain imaging research confirms that image and verbal cues associated with foods and beverages high in sugar produce higher preferences and higher emotional activation, making it harder for overweight people to resist eating unhealthy foods [252]. Several variables, including inflammation, oxidative stress, and insulin resistance, have been postulated to contribute to diet-induced brain damage, all of which can be affected by dietary consumption and are related to the onset of depression [48]. Maintaining a balanced diet with anti-inflammatory properties may aid in the prevention of depressive symptoms, particularly in men, smokers, and those who are inactive [253].

In observational studies, adherence to a healthy diet, particularly a traditional Mediterranean diet or avoidance of a pro-inflammatory diet, appears to have a protective effect against depression [16]. A population-based cohort study showed that people who adhered to a Mediterranean diet during midlife had a lower risk of depression later in life [16, 254]. Although the relationship between obesity and depression is known, there is little research on the clinical benefits of nutritional therapy for obese patients. A clinical study evaluating the effects of a traditional Brazilian diet (DieTBra) and extra virgin olive oil (EVOO) on anxiety and depressive symptoms in severely obese participants showed that both DieTBra and olive oil interventions were effective in reducing anxiety and depressive symptoms in severely obese adults and that these interventions could be combined with clinical protocols for the treatment of anxiety and depressive symptoms in severely obese patients [255].

Current evidence suggests that diet quality is a modifiable risk factor for affective disorders, however, further research is needed to investigate the impact of dietary patterns and weight loss on improving psychological symptoms. Rodriguez-Lozada et al. randomly assigned overweight and obese participants (n=305) to two low-calorie diets with different macronutrient distributions: a moderately high protein diet and a low-fat diet for 16 weeks to assess the effects of prescribed energy restriction on anxiety and depressive symptoms in overweight and obese participants, as well as some baseline potential predictive value of psychological characteristics for weight loss [256]. The nutritional intervention demonstrated beneficial effects of weight loss on trait anxiety scores in women, depression scores in all populations, and especially in women and subjects following a low-fat diet. In addition, weight loss can be predicted by anxiety status at baseline, which occurs primarily in women and those on a low-fat diet, and this trial suggests that weight loss triggers improvements in psychological traits, while anxiety symptoms predict those volunteers who benefit most from a balanced calorie restriction intervention, which would help to personalize precise nutrition [256]. In addition, a randomized clinical trial study by Hariri et al. demonstrated the beneficial effects of sumac (*Rhus coriaria L.*) supplementation with a calorie-restricted diet on anthropometric indices, oxidative stress, and inflammation in overweight or obese women with depression [257].

Probiotics have been demonstrated to have antidepressant responses and anti-inflammatory effects. Borges et al. conducted a systematic review and meta-analysis of overweight or obese patients to determine the effects of prebiotics on blood biomarkers of obesity, depression, and anxiety (including ACTH, cortisol, leptin, ghrelin, thyroid stimulating hormone (TSH), parathyroid hormone (PTH), vitamin D, and BDNF) [258]. Prebiotics were found to possibly facilitate the regulation of blood concentrations of ghrelin and CRP in overweight or obese individuals. A double-blind, randomized, placebo-controlled trial was conducted in obese men (n = 45) and women (n = 60), consisting of a 12-week weight loss period based on moderate energy restriction and a 12-week weight maintenance period [259]. Each subject consumed two capsules per day of either a placebo or probiotic supplement Lactobacillus rhamnosus CGMCC1.3724 (LPR) [259]. During both phases of the program, LPR supplementation increased weight loss in women, which was associated with an increased desire to eat on an empty stomach. In addition, the LPR female group showed a more pronounced decrease in food cravings as well as a decrease in Beck Depression Scale scores [259]. Significant benefits of LPR on fasting satiety and cognitive restraint were also observed in men [259]. These clinical observations support the hypothesis that the gut-brain axis may influence appetite control and related behaviors in obesity management. Hulkkonen et al. studied the efficacy of probiotics and/or fish oil in improving prenatal and postnatal depression and anxiety symptoms and found that diet quality was negatively associated with depressive symptoms in early pregnancy and 6 months postpartum and with anxiety symptoms in early pregnancy [260].

Probiotics, prebiotics, and mushroom extracts may be used to simultaneously prevent and treat obesity and depression, and further research is required to optimally use these substances in humans [30]. Furthermore, the using dietary interventions may demonstrate to be an appealing and cost-effective alternative or adjunctive treatment for the clinical management of these conditions. Prospective investigations of the connection between diet and mental health ought to use clearer definitions to define diet and include or control for significant confounding factors. Regulation of gut microbiota might be a new strategy for the treatment of both neuroinflammation and depression [30]. Biochemical markers are positively associated with the development and severity of obesity, depression as well as anxiety, and are regulated by changes in the composition of the gut microbiota [258]. The gut microbiota is a potentially important regulating pathway between diet and brain health. Altered gut microbiota may be a therapeutic strategy for depression and obesity. FMT is currently the most effective gut microbiota intervention [261]. FMT may be a promising therapeutic choice for neurological disorders, however, the available evidence remains sparse, with a limited number of human studies conducted or ongoing, and for some diseases, only animal trials have been conducted. To further elucidate the role of FMT in neurological disorders, large-scale double-blind randomized controlled trials are required [261].

**Figure 3:**
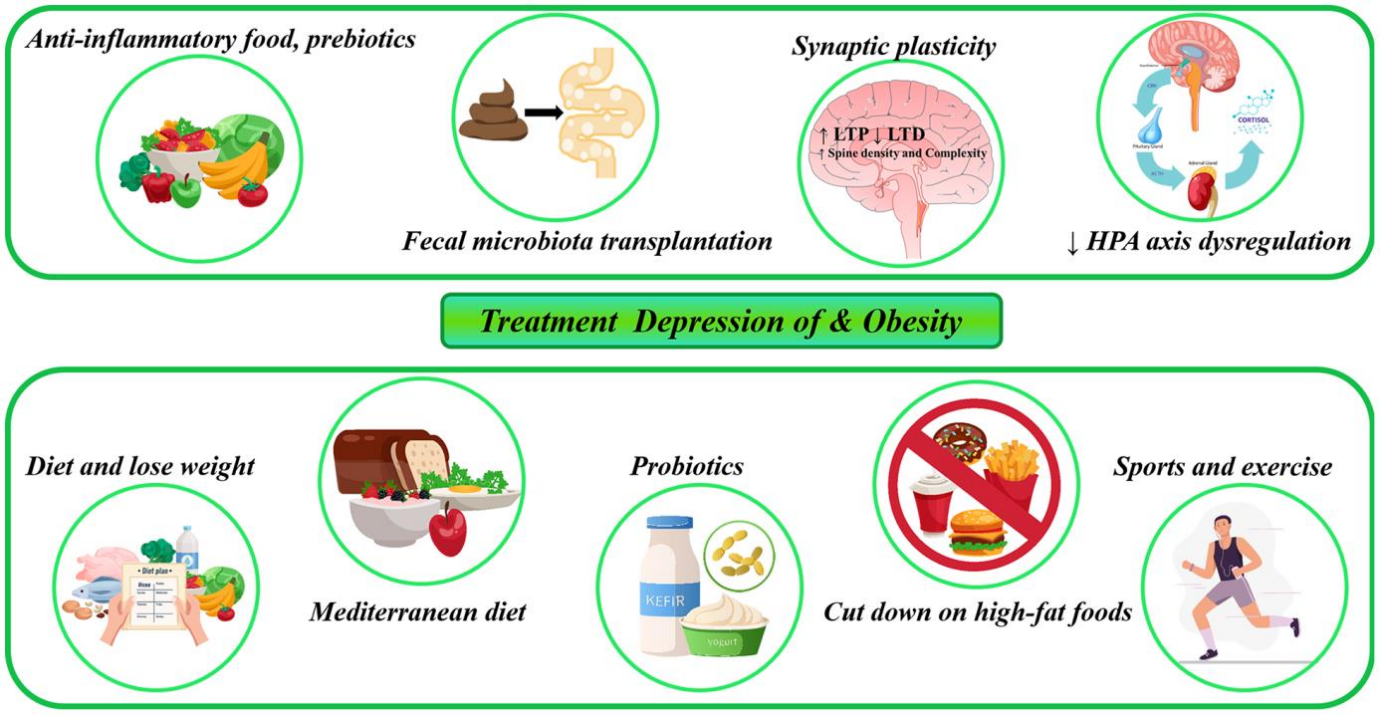
**Map of potential and possible future treatment options for depression and obesity comorbidity.** LTP, long-term potentiation; LTD, long-term depression; HPA axis, hypothalamo-pituitary-adrenal axis.

With the growing insight into the mechanisms underlying the complex interactions between diet and gut microbiota and their effects on depression, specific dietary patterns that aid in the prevention of anxiety and mood disorders may be identified [248]. Furthermore, preclinical studies have shown that exercise can improve various types of behaviors, such as depression and anxiety [262–264]. Morgan et al. showed that aerobic exercise had significant beneficial effects on depression-like, anxiety-like and cognitive-like behaviors during the healthy adult lifespan of C57BL/6 mice [265]. A combined treatment for both obesity and depression improves weight and mood risk factors more than treatment for each disease alone [266]. In a randomized controlled trial of 63 overweight/obese participants, Pilates and aerobic training were found to improve depression, anxiety levels and quality of life in overweight and obese people [267]. Recent clinical studies have shown that virtual reality exercise programs have a positive impact on BMI, depression levels, exercise enjoyment and exercise immersion in overweight middle-aged women. This is an effective home exercise program for people undergoing obesity management [268]. Developing tailored treatments according to a personalized medical approach to the biology of obesity-depression co-morbidities may ultimately benefit the patients involved. In Figure 3, we summarized the potential and possible future treatment options for obesity and depression (Figure 3).

## Overview

Acute/chronic inflammation, gut microbiota imbalance, gut-brain dysfunction, diminished neuroplasticity and HPA axis dysfunction are common mechanisms in the pathogenesis of depression, and several of these effects often co-occur in depression and also affect obesity at the same time. These factors would potentially provide a biologically based multi-level description of obesity and depression. The characterisation of the mechanisms of action of adipokines and lipokines and the identification of molecular targets of adipokines and lipokines will provide new ideas for this study of the co-morbidities of obesity and depression. Further study of adipokines and lipokines and their interaction with the brain may provide new therapeutic targets for the treatment of depression. Probiotics, herbal extracts and mushroom extracts may be used to prevent and treat the co-morbidities of obesity and depression. Modulation of the gut microbiota may be a novel strategy for the treatment of neuroinflammatory and depressive disorders. FMT may regain clinical attention as a therapy to restore gut flora for the clinical treatment of obesity and depression. The selection and development of treatments tailored to their biology, according to a personalised medicine approach, may ultimately benefit patients with depression and obesity.
